# Analysis of Self-Assembled Low- and High-Molecular-Weight Poly-L-Lysine–Ce6 Conjugate-Based Nanoparticles

**DOI:** 10.3390/biom14040431

**Published:** 2024-04-02

**Authors:** Minho Seo, Kyeong-Ju Lee, Bison Seo, Jun-Hyuck Lee, Jae-Hyeon Lee, Dong-Wook Shin, Jooho Park

**Affiliations:** 1 BK21 Program, Department of Applied Life Science, Konkuk University, Chungju 27478, Republic of Korea; 2 College of Biomedical and Health Science (RIBHS), Konkuk University, Chungju 27478, Republic of Korea

**Keywords:** photodynamic therapy, anticancer therapy, bioconjugate, peptide derivatives, nanoparticle

## Abstract

In cancer therapy, photodynamic therapy (PDT) has attracted significant attention due to its high potential for tumor-selective treatment. However, PDT agents often exhibit poor physicochemical properties, including solubility, necessitating the development of nanoformulations. In this study, we developed two cationic peptide-based self-assembled nanomaterials by using a PDT agent, chlorin e6 (Ce6). To manufacture biocompatible nanoparticles based on peptides, we used the cationic poly-L-lysine peptide, which is rich in primary amines. We prepared low- and high-molecular-weight poly-L-lysine, and then evaluated the formation and performance of nanoparticles after chemical conjugation with Ce6. The results showed that both molecules formed self-assembled nanoparticles by themselves in saline. Interestingly, the high-molecular-weight poly-L-lysine and Ce6 conjugates (HPLCe6) exhibited better self-assembly and PDT performance than low-molecular-weight poly-L-lysine and Ce6 conjugates (LPLCe6). Moreover, the HPLCe6 conjugates showed superior cellular uptake and exhibited stronger cytotoxicity in cell toxicity experiments. Therefore, it is functionally beneficial to use high-molecular-weight poly-L-lysine in the manufacturing of poly-L-lysine-based self-assembling biocompatible PDT nanoconjugates.

## 1. Introduction

Research on tumor-selective anticancer therapies, including photodynamic therapy (PDT), has been ongoing for decades [1,2]. Cancer is a fatal and intractable disease that is difficult to treat, and conventional chemotherapy with cytotoxic anticancer drugs has very little selectivity towards tumors, resulting in severe side effects [3]. Although various targeted anticancer therapies have been developed, only a few are effectively and widely used [4,5,6,7]. Among them, PDT is a method of inducing cell death used primarily for treating various cancers, utilizing photosensitizers, light of a specific wavelength, and oxygen [8,9]. Various types of PDT have been developed and are being used to fight the growing problem of antimicrobial resistance or treat different cancers such as pancreatic tumors, skin cancer and head and neck cancer [10,11,12,13]. In PDT, the excited photosensitizer directly reacts with cellular substrates to produce free radicals and reactive oxygen species (ROS). These reactive species or radicals interact with cellular components, causing damage to lipids, proteins, and nucleic acids, leading to cell death through necrosis or apoptosis [14,15]. ROS or singlet oxygen causes significant damage to cellular components, especially the lipids in cell membranes and mitochondrial membranes, leading to cell death. Using this principle, various PDTs such as vascular targeting PDT, cellular PDT, or upconversion-based PDT have been developed [16,17].

The combination of nanotechnology and PDT has made notable achievements in cancer therapy [18]. Traditionally, PDT agents have shown very poor physicochemical properties and present the problem of reaching only a small part of the tumor area that requires treatment [17]. However, with the recent integration of PDT agents and nanoparticle technologies, several new methods have been developed that significantly enhance the utility of PDT [19,20]. These PDT-based nanoparticles have demonstrated excellent functionality, improved tumor selectivity, and enhanced physicochemical properties, showing great potential as superior therapeutic agents [21,22]. One of the most commonly used PDT agents in nanoparticles is chlorin e6 (Ce6), which has been studied in combination with various nanomaterials [23].

One method of manufacturing PDT nanoparticles involves using peptides to form self-assembling nanoparticles [9,24]. Self-assembly is of particular interest in nanoparticle manufacturing, and it is not difficult to create self-assembling nanoparticles by using both PDT agents and peptides [25,26,27]. Through this method, many PDT agents have been developed and widely researched in preclinical studies. While countless combinations of peptides are possible, using poly-L-lysine allows for the utilization of its unique cationic nature and the manufacture of nanoparticles that internalize hydrophobic PDT agents [28,29]. Several types of poly-L-lysine-based nanoparticles have been manufactured by using this method, but effective and widely used poly-L-lysine-based PDT nanoparticles have yet to be developed.

**Figure 1:**
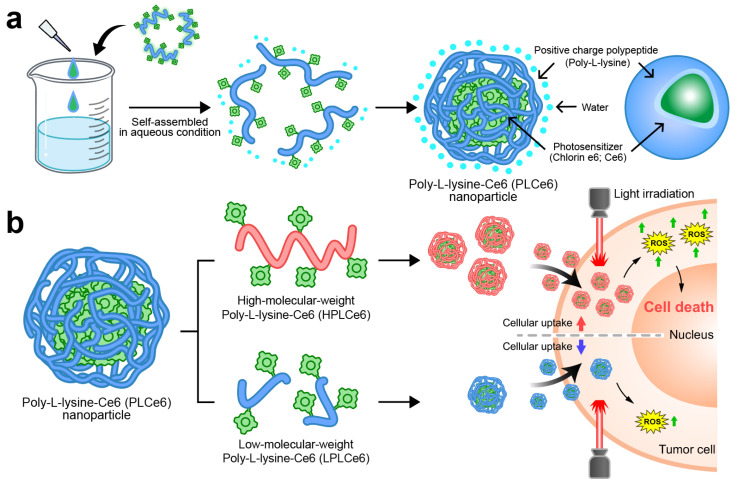
Schematic representation of PLCe6 PDT nanoparticles for cancer treatment through increased cellular uptake. (**a**) PLCe6 can form nanoparticles through self-assembly, based on its amphiphilic drug-based structure; (**b**) upon exposure to visible light, HPLCe6 can induce cytotoxicity due to high cellular uptake, whereas LPLCe6 struggles to induce cytotoxicity due to low cellular uptake.

In this study, we manufactured and evaluated two types of nanoparticles capable of self-assembly by using two different poly-L-lysine and Ce6 molecules (Figure 1). In particular, we proved through computer simulation how nanoparticles based on poly-L-lysine self-assemble amphiphilically in solvents. Initially, we synthesized and prepared low-molecular-weight poly-L-lysine molecules, while for high-molecular-weight poly-L-lysine (average molecular weight of 50 kDa), we used materials commonly available as reagents. The self-assembly of these two designed materials in aqueous solutions was evaluated through nanoparticle size and charge measurements. We also evaluated their effect on cell apoptosis and their absorption capabilities to compare which size of poly-L-lysine nanoparticles would be ideal for PDT therapy. This research will serve as a foundational study that can help in the clinical development of many PDT-based nanoparticles currently being developed.

## 2. Materials and Methods

### 2.1. Materials

Antibiotic/antimycotic solution (100×), acetonitrile (ACN), anhydrous dimethyl sulfoxide (DMSO), Dulbecco’s modified Eagle’s medium (DMEM), 1-ethyl-3-(3-dimethyl aminopropyl)carbodiimide (EDC), ethanol, L-lysine monohydrochloride (Lys), poly-L-lysine hydrobromide (HPL; average molecular weight of 50 kDa), *N*-hydroxysuccinimide (NHS), 2-(*N*-morpholino)ethanesulfonic acid monohydrate (MES monohydrate), phosphate-buffered saline (PBS), trifluoroacetic acid (TFA), and 2-(4-amidinophenyl)-6-indolecarbamidine dihydrochloride (DAPI) were purchased from Sigma-Aldrich (St. Louis, MO, USA). Fetal bovine serum (FBS) was obtained from Gibco (Waltham, MA, USA) and chlorin e6 (Ce6) was obtained from Leap Chem (Hong Kong). An EZ-cytox kit was obtained from DoGenBio (Seoul, Republic of Korea).

### 2.2. Preparation and Characterization of PL Nanoparticles

Initially, low-molecular-weight poly-L-lysine (LPL) was synthesized by utilizing the amino acid L-lysine with peptide coupling reagents. Specifically, L-lysine (Lys; 50 mg, 273.75 µmol), N-hydroxysuccinimide (NHS; 236.3 mg, 2.05 mmol), and 1-ethyl-3-(3-dimethyl aminopropyl)carbodiimide (EDC; 787.2 mg, 4.11 mmol) were sequentially dissolved in 2-morpholinoethanesulfonic acid (MES) buffer (5 mL, pH 5) and reacted for 24 h. Subsequently, the reaction was quenched by adding 1 N NaOH (1 mL) to adjust the pH to 10, followed by the addition of deionized water (DW) (4 mL) and lyophilization for 2 days. The freeze-dried mixture was then centrifuged at 3000 rpm for 5 min in ethanol to remove the unreacted EDC and NHS molecules, a process repeated four times. The residual organic solvent was removed by using a rotary evaporator, and the final product, LPL, was lyophilized for an additional 2 days. The molecular weight of the synthesized LPL (average molecular weight = 768.1 Da) was confirmed by using matrix-assisted laser desorption/ionization time-of-flight mass spectrometry (MALDI-TOF MS, AXIMA Performance, Kyoto, Japan) by ANYGEN (Gwangju, Republic of Korea).

Next, to fabricate nanoparticles that self-assemble in aqueous solutions, various ratios of Ce6 and LPL (LPL–Ce6 synthesis ratio 1:1, 1:2.5, or 1:5) and HPL (HPL–Ce6 synthesis ratio 1:10, 1:25, or 1:50) were conjugated by using the EDC/NHS reaction. For the synthesis of LPLCe6, LPL (15 mg, 19.53 µmol), Ce6 (11.65 mg, 19.52 µmol), NHS (6.74 mg, 58.56 µmol), and EDC (18.72 mg, 97.65 µmol) were dissolved in DW/DMSO (1:9 *v*/*v*, 6 mL). The solution was stirred at 25 °C for 24 h, purified by centrifugation in cold acetone at 4 °C at 3000 rpm, a step repeated three times, and then lyophilized to obtain LPLCe6 powder.

In the preparation of HPLCe6, HPL (15 mg, 0.3 µmol), Ce6 (1.79 mg, 3.00 µmol), NHS (1.04 mg, 9.04 µmol), and EDC (2.88 mg, 15.02 µmol) were dissolved in DW/DMSO (1:9 *v*/*v*, 6 mL) and stirred at 25 °C for 24 h. The mixture was then purified by centrifugation in cold acetone at 4 °C at 3000 rpm, a step repeated three times, and lyophilized to obtain HPLCe6 powder.

The conjugation of poly-L-lysine and Ce6 was verified by using UV–vis spectroscopy. Ce6, LPLCe6, and HPLCe6 were dissolved in anhydrous DMSO (1 mL), and their absorbance was measured with a SPECTROstar Nano spectrophotometer (BMG Labtech, Ortenberg, Germany). The purified LPLCe6 and HPLCe6 molecules were analyzed by using reverse-phase high-performance liquid chromatography (RP-HPLC) (1200 series, Agilent Technologies, Santa Clara, CA, USA). The RP-HPLC analysis utilized an Eclipse Plus C18 reverse-phase column (4.6 mm × 150 mm, 3.5 µm) with a gradient elution method. The mobile phase consisted of water with 0.1% trifluoroacetic acid (TFA) (90–10%) and acetonitrile with 0.1% TFA (10–90%), at a flow rate of 1 mL/min, with impurities detected by using a UV–vis detector at a wavelength of 405 nm.

### 2.3. Computer Simulation

Before the simulations, structures of LPLCe6 and HPLCe6 were generated by using the Pep-FOLD 4 website, producing a total of 20 structures each [30]. The structure with the optimal energy value was selected for further analysis. The structure in PDB format was then transferred to the Discovery Studio program, where the Ce6 structure was conjugated to the side chain of lysine. Subsequently, the structure of Lys–Ce6 was modeled with the PDB reader and manipulator in CHARMM-GUI. The combined structure was modeled by using the module for applying covalent bonds. The simulations were conducted by using the GROMACS 2021_2 program [31]. LPLCe6 and HPLCe6 were parameterized with the CHARMM-36m force field, and parameters were generated by using the CHARMM-GUI web server. Solvation was performed by using the Tip3 water model. Neutralization was achieved by adding chloride ions (Cl^−^) and sodium ions (Na^+^). A time step of 100 ns was employed, with a cutoff of 1.4 nm for short-range van der Waals and electrostatic interactions. Long-range electrostatics were computed by using the particle mesh Ewald method, employing a Fourier spacing of 0.24 nm and fourth-order interpolation. Bonds were constrained by using the LINCS algorithm. Rigid water temperature coupling utilized the v-rescale thermostat, while pressure coupling was managed by the Berendsen barostat during equilibration and the Parrinello−Rahman barostat during sampling. Simulations were conducted at 300 K and 1 bar. After the completion of the 100 ns MD simulation, the GROMACS energy analysis tool was employed to compare the values of Lennard-Jones and Coulomb interactions. These interactions were evaluated not only between solute and solvent but also between the solute molecules themselves. Furthermore, the resulting nanoparticles were imported into the Discovery Studio program in PDB format to analyze molecular interactions with the Analyze Trajectory tool. To evaluate the degree of aggregation for LPLCe6 and HPLCe6, the Protein Aggregation Analyzer in Discovery Studio was utilized, focusing on individual molecules.

### 2.4. Nanoparticle Analysis

The hydrodynamic size, distribution, and zeta potential of each PLCe6 nanoparticle ratio were measured after dissolving the substances in saline and saline containing 5% FBS at a concentration of 0.5 mg/mL, by using dynamic light scattering (DLS; Zetasizer Nano, Malvern Instruments, Worcestershire, UK). To indirectly verify the formation of particles under conditions mimicking the in vivo environment, we examined the fluorescence changes of LPLCe6 (1 mg/mL) and HPLCe6 (1 mg/mL) in the presence of various concentrations of NaCl (0–0.9%) by using a SpectraMax M2 microplate reader (Molecular Devices, San Jose, USA, λEx = 660 nm, λEm = 710 nm) (*n* = 5).

### 2.5. Cellular Uptake Study

To assess the endocytosis capability of LPLCe6 and HPLCe6 nanoparticles, murine colorectal carcinoma cells (CT26.WT) were seeded in a 35 mm confocal dish at a density of 5 × 10^4^ cells/well and cultured for 24 h in high glucose Dulbecco’s Modified Eagle’s Medium (DMEM) supplemented with 10% fetal bovine serum and 1% antibiotic/antimycotic solution. Subsequently, the cells were treated with LPLCe6 and HPLCe6 at a concentration of 10 µg/mL each and incubated at 37 °C for 3 h. Following incubation, the cells were washed once with phosphate-buffered saline (PBS) and fixed with 4% paraformaldehyde for 10 min. After fixation, cells were stained with 4’,6-diamidino-2-phenylindole (DAPI) in the dark for 10 min to visualize the nuclei. The intracellular localization of the nanoparticles was then imaged with an ECLIPSE Ti2 series microscope (Nikon, Tokyo, Japan).

### 2.6. In Vitro Cytotoxicity Assay of PLCe6 Nanoparticles

Cytotoxicity was assessed by using the EZ-cytox assay. The CT26.WT cells were seeded in a 96-well cell culture plate using high-glucose Dulbecco’s modified Eagle’s medium (DMEM) supplemented with 10% fetal bovine serum and 1% antibiotic/antimycotic solution, at a density of 1 × 10^4^ cells per well. The cells were allowed to stabilize for 3 h for attachment before treatment with Ce6, LPLCe6, or HPLCe6. Two independent experiments were conducted, one with laser irradiation and one without. In the experiment with laser irradiation, each well was irradiated with a 635 nm laser at an intensity of 20 mW/cm^2^ for 1 min, 2 h after treatment. Following an additional 12 or 24 h incubation period, the cells were washed twice with PBS. Subsequently, the cells were incubated in a DMEM medium containing 10% EZ-cytox solution for 1 h. The absorbance at 450 nm and 600 nm was measured with a SPECTROstar Nano spectrophotometer (BMG Labtech, Ortenberg, Germany) (*n* = 6).

### 2.7. Statistical Analysis

Statistical analysis was conducted using GraphPad Prism 9 (GraphPad Software 9.5.0). All experimental data were represented as mean ± standard deviation. A one-way ANOVA test was employed for comparison between two groups, and a p-value of less than 0.05 was considered statistically significant (* *p* < 0.05, ** *p* < 0.01, *** *p* < 0.001).

## 3. Results

### 3.1. Preparation of Chlorin e6-Conjugated Poly-L-Lysine (PLCe6) Nanoparticles

**Figure 2:**
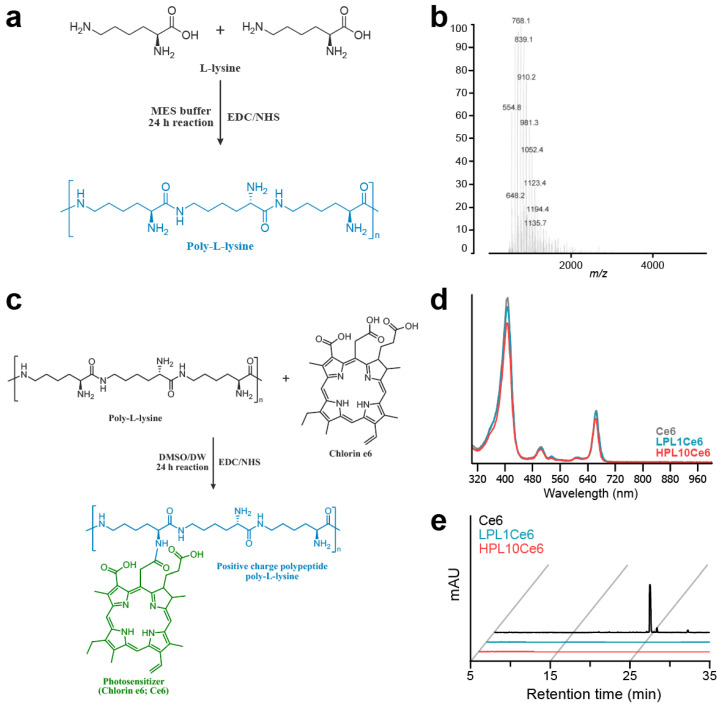
Preparation of LPL and PLCe6. (**a**) Schematic diagram of the structure and chemical synthesis of LPL; (**b**) measurement of the molecular weight of LPL with MALDI-TOF; (**c**) schematic diagram of the structure and chemical synthesis of PLCe6; (**d**) confirmation of the synthesis of LPL1Ce6 and HPL10Ce6 molecules from Ce6 with UV–vis spectroscopy after purification; (**e**) RP-HPLC of Ce6, LPL1Ce6 and HPL10Ce6.

We synthesized chlorin e6-conjugated poly-L-lysine (PLCe6) nanoparticles to explore the anticancer efficacy associated with the length of poly-L-lysine (PL). Initially, low-molecular-weight poly-L-lysine (LPL) was prepared through chemical conjugation by using the peptide coupling reagent EDC/NHS (Figure 2a). The molecular weight of the resultant LPL was confirmed via MALDI-TOF mass spectrometry (Figure 2b), which revealed an average molecular weight of 768.1 *m*/*z* [5Lys + K + 2Cl + H], indicative of the conjugation of five lysine units. Next, amide bonds were formed between the amine groups of PL and the carboxylic acid groups of Ce6 to achieve the conjugation of different lengths of PL with Ce6. The synthesis was carried out while maintaining the pH of the solution at approximately 6 (Figure 2c).

The chemical conjugation of PLCe6 was verified through UV–vis spectroscopy. The synthesized and purified LPL1Ce6 and HPL10Ce6 nanoparticles revealed absorption peaks at 663 or 662 nm (Figure 2d). This shift suggests a change in the molecular structure that affects electron distribution, thus altering the absorption spectrum. Finally, to confirm the complete conjugation of Ce6 to PL without any free Ce6 remaining, reverse-phase high-performance liquid chromatography (RP-HPLC) was employed, showing no detectable free Ce6 (Figure 2e).

### 3.2. Computer Simulation

**Figure 3:**
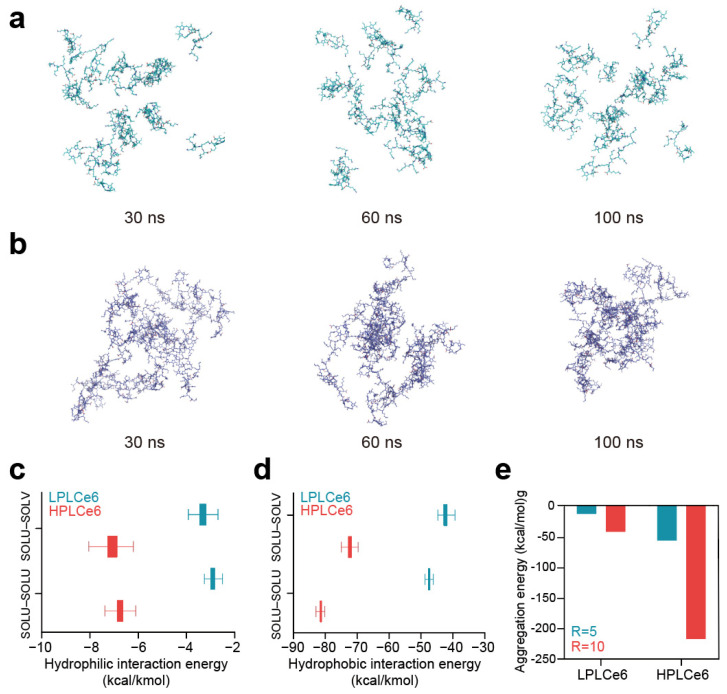
(**a**) LPLCe6 nanoparticle formulation during a 100 ns MD simulation; (**b**) HPLCe6 nano- particle formulation during a 100 ns MD simulation; (**c**) values for the hydrophilic (Coulomb) interacting energy during the MD simulations of LPLCe6 and HPLCe6; (**d**) hydrophobic (Lennard-Jones) interaction energy during the MD simulations of LPLCe6 and HPLCe6; (**e**) aggregation score for single molecules for HPLCe6 and LPLCe6.

In the comparative analysis between LPLCe6 and HPLCe6, it was beneficial to include details on the specific outcomes or insights gained from the in silico experiments. As time progressed, LPLCe6 and HPLCe6 underwent self-assembly in an ion-neutralized solvent model. Nanoparticle formation accelerated around 60 ns, with distinct clustering of nanoparticles observed from approximately 90 ns onwards. At the end of the 100 ns MD simulation, LPLCe6 did not form an assembled structure (Figure 3a). In contrast, HPLCe6 was constructed to form well-defined nanoparticle structures of approximately five molecules (Figure 3b).

LPLCe6 exhibited relatively low levels of particle cohesion and interaction energy compared to HPLCe6. This is attributed to HPLCe6 comprising 20 Lys amino acids and 4 Ce6 molecules, displaying sufficient amphiphilicity, whereas LPLCe6, composed of 1 Ce6 and 5 lysine molecules, shows relatively low hydrophobicity, making it difficult to exhibit amphiphilicity. Moreover, due to its sufficient molecular size, HPLCe6 can form a ‘theoretical’ particle as a single molecule. In contrast, LPLCe6, with its smaller molecular size, cannot form a particle shape as a single molecule and requires the aggregation of two or more molecules for interaction (Figure 3c). Even if interactions were formed, it was found that the gap between the hydrophobic and hydrophilic regions was too narrow for smooth particle formation. In summary, the analysis of nanoparticle morphology revealed that in the longer HPLCe6, the hydrophobic Ce6 portion formed a hydrophobic core within the HPLCe6 nanoparticle, while the hydrophilic lysine remained exposed outwardly, interacting with the solvent. Additionally, numerical values for the Lennard-Jones and Coulomb interactions were observed in 100 ns of MD simulation. Lennard-Jones energy is primarily attributed to van der Waals interactions such as London dispersion forces and radius absorption, categorizing them as hydrophobic interactions. Conversely, Coulomb energy mainly encompasses hydrogen bonding, ion interactions, etc., these being classified as hydrophilic interactions. Therefore, the interactions mediated by Ce6 in LPLCe6 and HPLCe6, representing hydrophobic interactions, and those influenced by lysine, indicating hydrophilic interactions, were described with such energy terms. 

The interaction energies between solvent and solute were also evaluated in a similar manner to consider the extent of solute–solvent interactions. As a result, the average Lennard-Jones interaction of HPLCe6 was lower than that of HPLCe6 (Figure 3d). The interparticle interaction of LPLCe6, measured at −2.90 Kcal/Kmol, was approximately 2.3 times lower than that of LPLCe6, which stood at −6.75. Additionally, the LPLCe6 interactions with solvent, characterized by LPLCe6 (−3.31), were lower compared to HPLCe6 (−7.07). Furthermore, the average Coulomb interactions of LPLCe6 were also weaker than those of HPLCe6 (Figure 3e). In terms of interparticle interactions, LPLCe6 exhibited values over 1.7 times lower than HPLCe6, with LPLCe6 (−47.45) against LPLCe6 (−81.04). Similarly, solvent–solute interactions showcased values over 1.7 times lower for LPLCe6 (−42.31) compared to HPLCe6 (−72.21). Only HPLCe6 could theoretically form a nanoparticle from a single molecule, accelerating nanoparticle formation. The presence of Lys–Ce6 facilitated aggregation of the poly-L-lysine structure, confirmed through calculations of aggregation scores by using the Analyze Protein Aggregation module. In single molecules, LPLCe6 with only one Ce6 molecule had an average aggregation score of −0.176 for four lysines, whereas HPLCe6 with four Ce6 molecules had a score of −0.619, indicating increased energy due to Ce6’s hydrophobicity contrasting with the hydrophilicity of lysine.

### 3.3. Characterization of PLCe6 Nanoparticles

**Figure 4:**
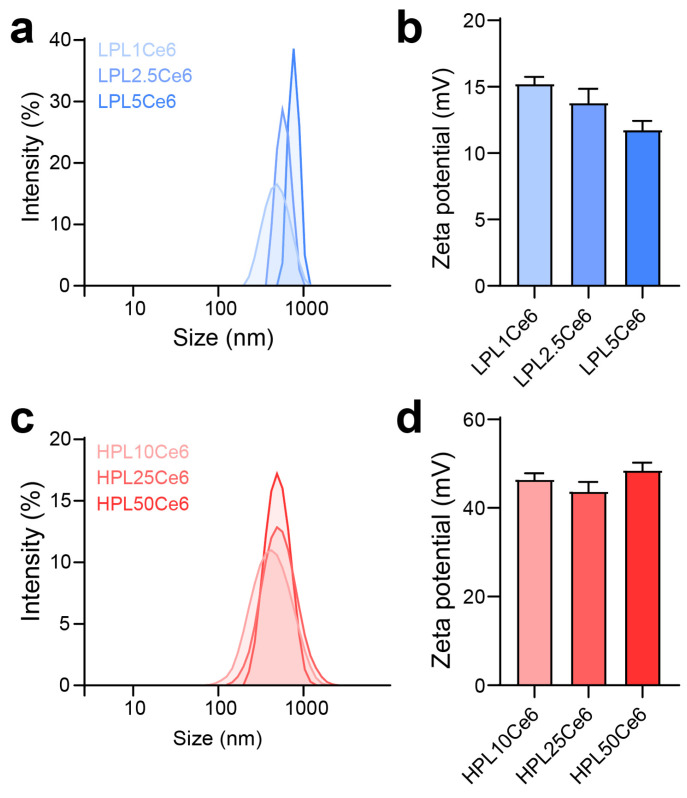
Optimization preparation of PLCe6. (**a**) Size distribution of LPL1Ce6, LPL2.5Ce6, and LPL5Ce6 confirmed through DLS. Results are presented as mean ± SD (*n* = 3). (**b**) Zeta potential of LPL1Ce6, LPL2.5Ce6, and LPL5Ce6. Results are shown as mean ± SD (*n* = 3). (**c**) Size distribution of HPL10Ce6, HPL25Ce6, and PL50Ce6 confirmed through DLS. Results are presented as mean ± SD (*n* = 3). (**d**) Zeta potential of HPL10Ce6, HPL25Ce6, and HPL50Ce6. Results are shown as mean ± SD (*n* = 3).

The optimization of LPLCe6 and HPLCe6 nanoparticles was conducted across various ratios to assess their size, stability, and zeta potential. Due to the amphiphilic nature resulting from the conjugation of the cationic peptide (PL) with the hydrophobic drug (Ce6), PLCe6 nanoparticles demonstrated stability in saline. The diameter of LPLCe6 nanoparticles increased with the amount of Ce6 conjugated to LPL. Specifically, diameters for the ratios (LPL1Ce6, LPL2.5Ce6, and LPL5Ce6) were measured at 474.1 ± 44.1 nm, 789.3 ± 210.7 nm, and 1337 ± 292 nm, respectively, indicating an increase in both diameter and distribution (Figure 4a). Concurrently, the zeta potential showed a gradual decrease to 15.31 ± 0.45, 13.87 ± 1.09, and 11.84 ± 0.62 for each respective ratio, suggesting challenges in nanoparticle formation control due to increased hydrophobic interactions among Ce6 molecules as the Ce6 conjugation ratio rose (Figure 4b). For further experiments, LPL1Ce6 was selected for its relatively higher particle stability and denoted simply as LPLCe6.

In contrast, HPLCe6 nanoparticles, across ratios (HPL10Ce6, HPL25Ce6, HPL50Ce6), exhibited diameters of 359.87 ± 37.44 nm, 465.20 ± 32.57 nm, and 481.10 ± 42.07 nm, respectively, showing a gradual increase in diameter with a narrow distribution (Figure 4c). The zeta potentials remained comparatively stable at 46.73 ± 1.24, 44.03 ± 2.15, and 48.75 ± 1.51 for each ratio, indicating that the increase in hydrophobicity ratio did not compromise stability in aqueous solutions, despite the particle size increase (Figure 4d). The smallest nanoparticles, designated as LPL1Ce6 or HPL10Ce6, were LPLCe6 or HPLCe6, respectively. To verify potential particle formation within the body, we additionally measured the sizes of LPLCe6 and HPLCe6 nanoparticles in saline containing 5% FBS. As a result, the average size of LPLCe6 nanoparticles was 1027.1 ± 212.8 nm, while that of HPLCe6 nanoparticles was significantly smaller at 350.8 ± 119.0 nm (Figure S1). These results suggest that HPLCe6 nanoformulates reach an appropriate size in the body, compared with LPLCe6. These results demonstrate that HPLCe6 showed higher particle stability compared to LPLCe6, demonstrating advantageous physicochemical properties for nanoparticle formation.

### 3.4. Analysis of PLCe6 Nanoparticles

**Figure 5:**
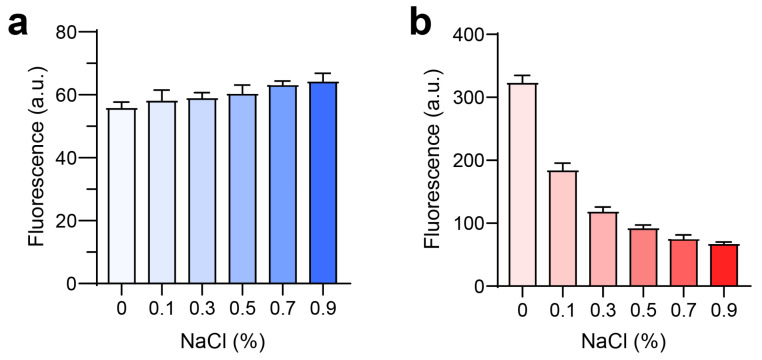
Analysis of PLCe6 nanoparticles under conditions similar to an in vivo environment. (**a**,**b**) Fluorescence intensity of (**a**) LPLCe6 and (**b**) HPLCe6 in an environment similar to in vivo conditions. Results are presented as mean ± SD (*n* = 5).

Further investigation into the potential for nanoparticle formation in vivo was conducted by examining the effect of NaCl on fluorescence intensity. For LPLCe6, an increase in NaCl concentration (from 0% to 0.9%) did not significantly alter the fluorescence intensity (Figure 5a). However, HPLCe6 exhibited a notable decrease in fluorescence intensity with increasing NaCl concentrations (Figure 5b). These results indicate that the low fluorescence intensity of nanoparticles in saline is due to a quenching effect resulting from the reduced intermolecular distances among Ce6 molecules. These data suggest that PLCe6 molecules may form nanoparticles by themselves in vivo.

### 3.5. Tumor Cell Uptake and Cytotoxicity of PLCe6 Nanoparticles In Vitro

**Figure 6:**
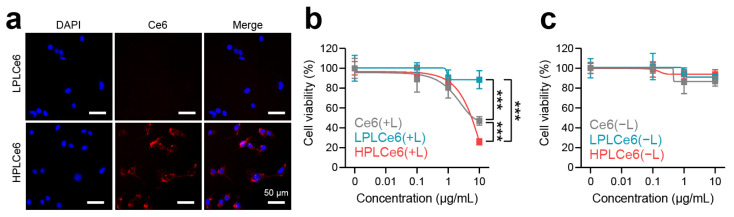
Cellular uptake and cytotoxicity effects of PLCe6 nanoparticles. (**a**) Cellular uptake of PLCe6 nanoparticles in CT26.WT cells treated for 3 h was observed by using fluorescence microscopy (red; Ce6, λEx = 540 nm, λEm = 605 nm, blue; DAPI, λEx = 375 nm, λEm = 460 nm); scale bars = 50 µm for all. (**b**) Cytotoxicity evaluation of Ce6, LPLCe6, and HPLCe6 on CT26.WT cells for 24 h with laser irradiation; (**c**) without laser irradiation (*n* = 6), mean ± SD, *** *p* < 0.001.

Murine colorectal carcinoma (CT26.WT) cells are widely used cells derived from BALB/c mice and have been receiving attention recently along with cancer immunotherapy; hence, cellular experiments related to cells were conducted using CT26.WT cells. To assess nanoparticle uptake by CT26.WT tumor cells, LPLCe6 and HPLCe6 nanoparticles were administered at a concentration of 10 µg/mL and observed after 3 h. Remarkably, while LPLCe6 nanoparticles exhibited no detectable fluorescence from Ce6 molecules, indicating minimal cell uptake, HPLCe6 nanoparticles demonstrated significant fluorescence intensity within the cytosol (Figure 6a). This observation is attributed to the stronger positive charge of HPLCe6 compared to LPLCe6, indicating that HPLCe6 nanoparticles exhibit superior cellular uptake capabilities relative to LPLCe6. Subsequent analysis focused on the cytotoxic effects of these nanoparticles post laser irradiation. At a concentration of 10 µg/mL, HPLCe6 nanoparticles induced significant cytotoxicity, comparable to that caused by LPLCe6 nanoparticles. LPLCe6 nanoparticles, despite laser irradiation, failed to induce substantial cell apoptosis, likely due to their lower cellular uptake attributed to their weak positive charges. This insufficient uptake, in turn, did not produce significant levels of ROS within the cells, leading to inducing low substantial cell death. Conversely, HPLCe6 nanoparticles, with their strong positive charges, successfully generated intracellular ROS owing to their high uptake by cells, thus inducing cell death (Figure 6b and Figure S2a). No significant cytotoxic effect of them was observed under dark conditions (Figure 6c and Figure S2b). Their phototherapeutic index (PI) was analyzed (Table S1) and the results show that the PI value of HPLCe6 is higher than Ce6. The effectiveness of PDT shown may not be significantly different before and after nanoparticle formation, but, in general, nanoparticle formation will contribute significantly to the improvement of tumor targeting and therapeutic effects [9,22]. These findings underline the enhanced cell uptake and apoptosis-inducing capabilities of HPLCe6 nanoparticles over LPLCe6, highlighting the potential therapeutic advantages of HPLCe6 in targeted cancer treatments.

## 4. Discussion

The use of nanomedicine-based photodynamic therapy (PDT) is one of the interesting fields that modern science is paying great attention to, especially with regard to tumor treatment. The clinical use and application of PDT to patients are fraught with various technical challenges, but self-assembling PDT nanoparticles show potential in overcoming these issues [8,32,33]. From this perspective, several peptides and PDT agent-based substances have been synthesized and developed, including poly-L-lysine and PDT conjugates [34,35]. Poly-L-Lysine-based biomolecules with PDT were synthesized a few years ago and evaluated in various forms with therapeutic potentials. The synthesized lysin and PDT conjugates showed different cell absorption and cellular effects depending on the charge, and showed tumor targeting effects in animals [36,37,38]. Therefore, in this study, the authors have focused on demonstrating how the poly-L-lysine and PDT-based nanoparticles can form and function by using high- and low-molecular-weight poly-L-Lysine and Ce6 conjugates. As a result, this study is the first to show the formation of self-assembling nanoparticles of lysine and Ce6 through computer simulations, among other methods, and observed the conditions of particle formation through various methods. The combination of positively charged lysine and the hydrophobic Ce6 demonstrated successful nanoparticle formation.

In this paper, we prepared and comparatively evaluated particles based on lysine and Ce6 of two different molecular weights. Initially, the authors hypothesized that materials based on low-molecular-weight lysine would be superior due to its compact molecular size. This was because peptides with excessively large molecular weights may limit the functionality as PDT and hinder self-assembled nanoparticle formation in solution. However, the experimental results with LPLCe6 and HPLCe6 nanoparticles showed that the high-molecular-weight Lysine-Ce6 conjugates (HPLCe6 nanoparticles) exhibited better self-assembled nanoparticle formation by themselves. The difference in physicochemical properties was also reflected in cellular absorption and functionality, ultimately indicating that high-molecular-weight conjugates exhibited stronger efficacy as PDT agents. Finally, the phototherapeutic index (PI) using different types of photosensitizers in this study was analyzed and summarized. We presented their therapeutic potential and property for PDT through the analysis of PI for Ce6 and PLCe6 [39]. This research could influence further studies on the formation and evaluation of self-assembled peptide and drug-based nanoparticles for therapy.

## 5. Conclusions

In this study, two types of poly-L-lysine were prepared and subsequently evaluated concerning their complexation with chlorin e6 (Ce6). The low-molecular-weight poly-L-lysine was synthesized separately, and both peptides were directly chemically bound to Ce6, a PDT agent. As a result, both PDT materials successfully formed self-assembling nanoparticles based on amphiphilicity. Computer simulations were used to predict and evaluate this self-assembly, allowing for an understanding of the self-assembly mechanisms of LPLCe6 and HPLCe6 molecules. While both materials formed particles, the high-molecular-weight lysine-based HPLCe6 nanoparticles showed slightly superior cell permeability and cytotoxicity. This research could significantly influence future studies on the manufacturing of self-assembling PDT nanoparticles.

## Data Availability

Data is contained within the article.

## Supplementary Materials

The following supporting information can be downloaded at https://www.mdpi.com/article/10.3390/biom14040431/s1, Figure S1: the size distribution of HPLCe6 and LPLCe6 nanoparticles, Figure S2: cytotoxicity evaluation of Ce6, LPLCe6, and HPLCe6 on CT26.WT cells for 12 h (*n* = 6), mean ± SD, *** *p* < 0.001, Table S1: photo- and cytotoxicity of Ce6, LPLCe6 and HPLCe6 expressed by IC50 values of the mean ± SD (µg/mL).
