# Supplementary material for: Analysis of Self-Assembled Low- and High-Molecular-Weight Poly-L-Lysine–Ce6 Conjugate-Based Nanoparticles
Source: Biomolecules. 2024 Apr 2;14(4):431. doi: 10.3390/biom14040431 (PMC11048146; doi:10.3390/biom14040431)
Supplement: Supplementary file 1 [file biomolecules-14-00431-s001.zip › biomolecules-2906456-supplementary.pdf]

Supporting Information

Analysis of Self-Assembled Low- and High-Molecular-Weight Poly-L-Lysine-Ce6 Conjugate-Based Nanoparticles

Minho Seo[1†], Kyeong-Ju Lee[1†], Bison Seo[2], Jun-Hyuck Lee[1], Jae-Hyeon Lee[1], Dong Wook Shin[2], and Jooho Park[1,2*]

[1]Department of Applied Life Science, BK21 Program, Konkuk University, Chungju 27478, Republic of Korea

[2]College of Biomedical and Health Science (RIBHS), Konkuk University, Chungju 27478, Republic of Korea

[†]These authors contributed equally to this work.

***Corresponding author:** Jooho Park

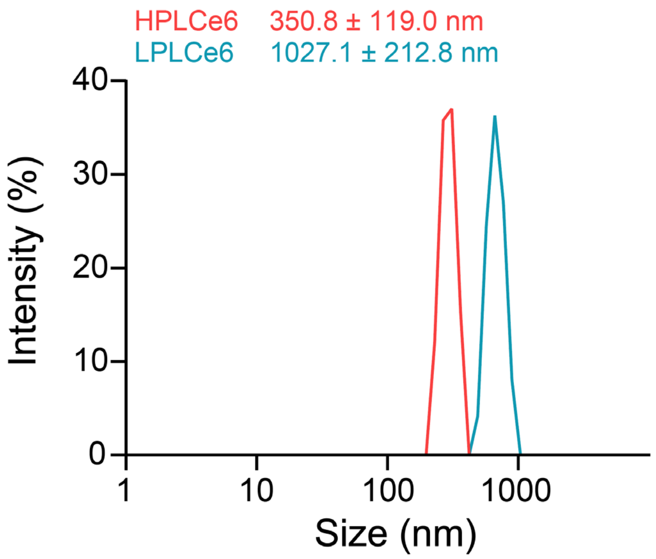

Figure S1. The size distribution of HPLCe6 and LPLCe6 nanoparticles

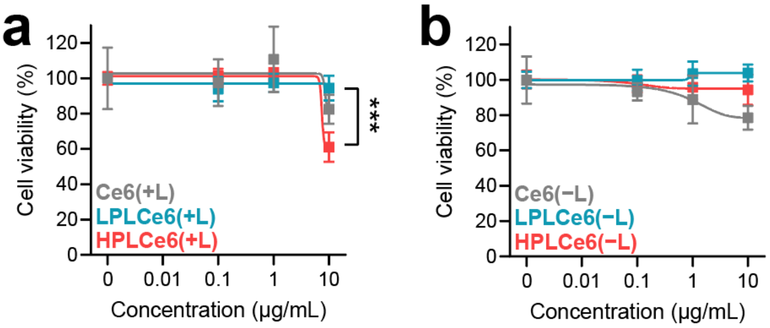

Figure S2. Cytotoxicity evaluation of Ce6, LPLCe6, and HPLCe6 on CT26.WT cells for 12 hours (a) with laser irradiation, and (b) without laser irradiation ($n = 6$), mean ±SD, $^{***}p < 0.001$.

Table S1. Photo- and cytotoxicity of Ce6, LPLCe6 and HPLCe6 expressed by IC50 values of the mean ± SD (μg/mL).

Cell line	Compounds	Cytotoxicity	Phototoxicity	PI*
CT26.WT	Ce6	83.30 ± 47.70	8.14 ± 3.04	10.23
	LPLCe6	243.84 ± 194.25	126.23 ± 87.48	1.93
	HPLCe6	350.08 ± 274.22	4.34 ± 0.84	80.66

*Phototherapeutic index (PI) = Cytotoxicity/Phototoxicity
